# Understanding CBHI hospitalisation patterns: a comparison of insured and uninsured women in Gujarat, India

**DOI:** 10.1186/1472-6963-14-320

**Published:** 2014-07-26

**Authors:** Sapna Desai, Tara Sinha, Ajay Mahal, Simon Cousens

**Affiliations:** 1 Faculty of Epidemiology and Population Health, London School of Hygiene and Tropical Medicine, London, UK; 2 Self Employed Women’s Association, Ahmedabad, Gujarat, India; 3 School of Public Health and Preventive Medicine, Monash University, Melbourne, Australia

**Keywords:** Community-based health insurance, Treatment-seeking behaviour, Health insurance, India, Hysterectomy, Female, Hospitalization

## Abstract

**Background:**

Community-based health insurance has been associated with increased hospitalisation in low-income settings, but with limited analysis of the illnesses for which claims are submitted. A review of claims submitted to VimoSEWA, an inpatient insurance scheme in Gujarat, India, found that fever, diarrhoea and hysterectomy, the latter at a mean age of 37 years, were the leading reasons for claims by adult women. We compared the morbidity, outpatient treatment-seeking and hospitalisation patterns of VimoSEWA-insured women with uninsured women.

**Methods:**

We utilised data from a cross-sectional survey of 1,934 insured and uninsured women in Gujarat, India. Multivariable logistic regression identified predictors of insurance coverage and the association of insurance with hospitalisation. Self-reported data on morbidity, outpatient care and hospitalisation were compared between insured and uninsured women.

**Results:**

Age, marital status and occupation of adult women were associated with insurance status. Reported recent morbidity, type of illness and outpatient treatment were similar among insured and uninsured women. Multivariable analysis revealed strong evidence of a higher odds of hospitalisation amongst the insured (OR = 2.7; 95% ci. 1.6, 4.7). The leading reason for hospitalisation for uninsured and insured women was hysterectomy, at a similar mean age of 36, followed by common ailments such as fever and diarrhoea. Insured women appeared to have a higher probability of being hospitalised than uninsured women for all causes, rather than specifically for fever, diarrhoea or hysterectomy. Length of stay was similar while choice of hospital differed between insured and uninsured women.

**Conclusions:**

Despite similar reported morbidity patterns and initial treatment-seeking behaviour, VimoSEWA members were more likely to be hospitalised. The data did not provide strong evidence that inpatient hospitalisation replaced outpatient treatment for common illnesses or that insurance was the primary inducement for hysterectomy in the population. Rather, it appears that VimoSEWA members behaved differently in deciding if, and where, to be hospitalised for any condition. Further research is required to explore this decision-making process and roles, if any, played by adverse selection and moral hazard. Lastly, these hospitalisation patterns raise concerns regarding population health needs and access to quality preventive and outpatient services.

## Background

Community based health insurance (CBHI) is a health financing arrangement that aims to reduce risk of catastrophic health expenditure and improve access to health care in low-income settings. Most CBHI schemes are rooted, to varying degrees, in principles of risk-sharing, community solidarity, participatory decision-making and voluntary affiliation [1]. According to findings from a systematic review in 2009 that covered 31 studies and 118 schemes, and an earlier review of 12 schemes in Asia and Africa, there is strong evidence that CBHI schemes can decrease out of pocket spending on health care [2,3] A large proportion of schemes in low-income settings cover only inpatient hospitalisation expenses, and CBHI coverage has been associated with increased hospitalisation in a number of studies [4-9]. However, health systems issues such as poor quality of services and lack of patient empowerment remain barriers to increased treatment-seeking [10,11], while low enrolment limits coverage of CBHIs and similar voluntary schemes [12]. Despite these weaknesses, CBHIs continue to be implemented in many low-income countries, as a potential tool to improve access and financial security [1].

Much of the existing research on CBHIs and hospitalisation in low-income settings has focused on the association with increased hospitalisation, with limited analysis of the underlying health conditions that drive utilisation. In fact, careful analysis of morbidity profiles in combination with treatment-seeking behaviour by (CBHI) insurance status can shed light on the role of insurance in increasing use of both outpatient and inpatient health services, including the risk of inefficient provider-induced or patient overutilization of services (moral hazard). Analysis of data on morbidity and treatment patterns can also be useful for exploring whether there is a higher likelihood of enrolment by persons more prone to seeking care (adverse selection).

To our knowledge, only three CBHI studies have integrated an epidemiological analysis to compare hospitalisation amongst the insured and uninsured in low-income settings. Devadasan et al. compared reported illness – categorised as minor, major or chronic – amongst matched insured and uninsured households in rural India [13]. Despite similar levels of minor and chronic illness, the insured were 2.5 times more likely to present with a major ailment and almost twice as likely to be hospitalised as the uninsured. However, the insurance scheme covered only hospitalisation and all hospitalisations were automatically categorised as major illness – which may account for the difference in reported morbidity patterns between the insured and uninsured. In contrast, an evaluation of Filipino micro-insurance units that cover inpatient care reported similar incidence of recent morbidity amongst the insured and uninsured. Yet the insured had a 50% higher risk of hospitalisation for both communicable and non-communicable illnesses, as well as more physician encounters and institutional deliveries [14]. Similarly, a detailed study of the impact of the Bwamanda hospital scheme in Zaire reported a 2.9-fold higher admission rate for the insured [15]. The authors analysed care for two ‘justified high priority’ conditions – caesarean sections and strangulated hernias – and found significantly lower rates amongst the uninsured.

In our own experience at VimoSEWA, a CBHI in India that covers only 24-hour or longer hospitalisation, a recent analysis of claims indicated that close to forty percent of adult hospitalisation was for common, typically mild illnesses such as fever and diarrhoea, as well as hysterectomy amongst women in their mid-thirties [16]. This pattern surprised VimoSEWA’s management and SEWA’s community health team, who questioned if: (i) the scheme’s inpatient-only design effectively served to replace outpatient treatment for common illnesses such as diarrhoea and fever with reimbursable, inpatient hospitalisation (ii) the scheme and/or providers promoted unnecessary procedures, particularly hysterectomy at a young age (iii) some of the burden of illness and hospitalisation was preventable through community intervention. As a first step in exploring the first two questions, we conducted a household survey to compare morbidity, outpatient treatment-seeking and hospitalisation patterns of women insured by VimoSEWA with uninsured women in the same geographical areas. A health education intervention was designed to test (iii), along with qualitative research on the three questions. This paper reports the findings from the household survey.

### VimoSEWA

VimoSEWA is a voluntary, community-based insurance scheme initiated in 1992 by the Self-Employed Women’s Association (SEWA), a women’s trade union with 1.3 million members in nine states of India [17]. The majority of VimoSEWA’s members are women workers in the informal sector in the state of Gujarat. In partnership with insurance companies, VimoSEWA promotes a range of voluntary insurance products to women workers through its non-profit cooperative. VimoSEWA insures adult women as the primary insured, who have the option to purchase additional coverage for spouses and children (Table 1).

**Table 1:** VimoSEWA Health Products (Indian Rupees. 1 USD = INR 54.5)

	**Member**	**Spouse**	**Children**	**Total**
	**Scheme 1**			
**Annual premium**	175	125	100	400
**Annual total hospitalisation coverage**	2,000	2,000	2,000	6,000
	**Scheme 2**			
**Annual premium**	375	350	100	825
**Annual total hospitalisation coverage**	6,000	6,000	2,500	14,500

Like most Indian CBHI schemes [18], VimoSEWA provides hospitalisation coverage that includes hospital and provider charges, medicines, transportation and other expenditure incurred while admitted in an inpatient facility. For a claim to be admissible under the scheme, the member must be hospitalised for a minimum of 24 hours. VimoSEWA does not cover expenditure on outpatient treatment or childbirth. In 2012, the health scheme insured approximately 80,000 policies, about 6 percent of SEWA’s membership [19]. No other micro- or community-based health insurance schemes operated in VimoSEWA’s coverage areas at the time of this survey and very few informal sector households (in Gujarat and India) hold any other voluntary private health insurance policies^a^. A subsidized government health insurance scheme, the Rashtriya Swasthya Bima Yojana (RSBY), began roll-out in SEWA’s focus rural areas in 2011 [20].

Payment for hospitalisation costs is made in two ways. In Ahmedabad city and parts of rural Gujarat, members can obtain ‘cashless’ treatment if they are admitted in empanelled public, private for profit and private non-profit hospitals (the latter are locally known as trust hospitals). Admitted members inform VimoSEWA as soon as they are admitted and VimoSEWA pays the hospitals directly. In other areas, members pay out-of-pocket and are reimbursed for expenses on submission of hospital bills.

Previous research at VimoSEWA has found that the scheme provides members with a degree of financial protection, but the coverage is not comprehensive [21]. Twenty-three percent of VimoSEWA members hospitalised in 2003 experienced catastrophic health expenditure, defined as annual hospital expenditure greater than 10% of annual income, after reimbursement [22]. The scheme has also been examined from an equity perspective. Research findings indicated that the scheme was successful in enrolling the poor, and utilisation patterns were broadly comparable among urban members of different socioeconomic groups. Among rural members however, the better off were more likely to submit claims. Barriers to utilisation included distance to hospitals, difficulty with claims paperwork and lack of awareness about insurance coverage [23]. Child care, household responsibilities and opportunity costs such as lost wages were also identified as obstacles to treatment-seeking, for women in particular [24].

A review of VimoSEWA health claims in 2001 indicated that the most common reasons for adult hospitalisation were accidents, malaria, gastroenteritis and hysterectomy [21]. A follow-up review in 2009 revealed that the leading reasons for adult hospitalisation claims were for illnesses such as fever, diarrhoea/gastroenteritis and respiratory infection – which are considered common illnesses amenable to prevention or outpatient treatment if diagnosed early. Hysterectomy was the primary reason for claims amongst rural women, at an average age of 37 years, considerably younger than in countries where data are available [25-28]. A survey conducted by Ranson in 2000 amongst 242 VimoSEWA-insured and 381 uninsured households did not find evidence of increased hospitalisation amongst insured women [29]. Since then, VimoSEWA has not assessed hospitalisation rates or compared treatment-seeking of insured members with the uninsured.

#### Study objectives

This analysis is one of a set of studies at SEWA to explore treatment-seeking behaviour amongst low-income women in Ahmedabad city and district in Gujarat. It builds on previous research at VimoSEWA by comparing insured women to the uninsured, and contributes to the literature on CBHI by integrating an epidemiological approach to the analysis of healthcare utilisation patterns. We examined three issues. First, we examined demographic characteristics of VimoSEWA-insured and uninsured women to identify factors associated with insurance coverage, particularly those which could potentially affect treatment-seeking. Second, we compared insured and uninsured women with respect to the prevalence of morbidity in the past month and place where treatment was sought (self/outpatient clinic/hospital/none), in order to examine treatment choices that are not covered by VimoSEWA’s inpatient-only scheme. Third, we compared insured and uninsured women with respect to hospitalisation in the past six months, comparing type of illness, length of stay and place of hospitalisation.

## Methods

This study utilised data from a cross-sectional baseline household survey conducted from January to March 2010 amongst a sample of insured and uninsured households in Ahmedabad district and Ahmedabad city, Gujarat. The survey was designed to provide baseline information for a subsequent evaluation of a health education intervention amongst insured and uninsured women. We compared demographic, morbidity and treatment-seeking patterns across 28 clusters where the intervention was to be implemented. The survey was conducted in 16 rural and 12 urban clusters, with clusters defined as discrete geographical units serviced by a single SEWA community health worker (CHW). CHWs serve both insured and uninsured households: approximately eight to ten percent of the 200–500 households in each cluster are insured by VimoSEWA. The sample was stratified by urban and rural location, as urban rates of claim submission have been established to be higher than in rural areas in two previous analyses [16,23].

### Household selection

For insured households, 35 households from each cluster were randomly selected from the VimoSEWA database. A researcher followed each CHW on her daily rounds to list uninsured households, from which 35 were also randomly selected. Thus, 70 households were selected per cluster to give a total of 1,960 households^b^.

### Data collection

The survey collected information for all family members on demographic and socioeconomic characteristics, morbidity and all treatment-seeking behaviour in the past 30 days and hospitalisation and associated expenditure in the past six months. In each household, an adult woman was selected for interview. In insured households, the respondent was the primary VimoSEWA policy holder. In uninsured households, the primary SEWA union member or spouse of the male head of household was selected. All respondents provided oral informed consent. Both the local ethics committee and CHWs considered this the most appropriate convention, rather than written consent, as most women in the area have not attended formal schooling. Ethics approval was granted by the Executive Committee of the SEWA Health Cooperative.

### Data analysis

Data were entered into a Microsoft Access database and analysed using Stata 11. The svyset command was utilised to take into account the cluster sampling, sampling weights for insured and uninsured households, and the rural/urban stratification. Sampling weights were defined by cluster, as both the population size and penetration of insurance varied by CHW work area. All tables present weighted proportions.

We conducted three analyses to compare insured and uninsured women. In the first analysis, we examined demographic and socioeconomic characteristics of insured and uninsured households and women in order to identify any factors that may later be associated with differences in treatment-seeking behaviour. Socioeconomic indicators (income, education, dwelling type, toilet, drinking water access) are presented and analysed separately rather than as a score derived using principal component analysis, as we believed that some of these variables could be independently associated with morbidity or hospitalisation. Although women workers in the informal economy typically engage in multiple income-earning activities [30], only the respondent’s stated primary occupation was included in the analysis. After examining unadjusted odds ratios calculated using logistic regression, multivariable logistic regression was used to identify predictors of insurance status. We included variables observed to be associated with (p ≤ .05) or those that could be theoretically associated with, insurance coverage. Results are presented as adjusted odds ratios with 95% confidence intervals. Overall p-values for variables with more than two levels were obtained using Wald tests. Urban and rural data were stratified in crude analyses and then combined in multivariable analyses, with location formally tested for effect modification.

In the second set of analyses, data on recent morbidity and treatment-seeking were compared between insured and uninsured women. Recent morbidity was defined as any illness episode in the past month, to limit recall bias and to capture outpatient treatment-seeking behaviour as accurately as possible. Morbidity in the past 30 days included chronic illness, as we did not inquire about chronic illness separately at the individual level.

In the third analysis, the association between current insurance coverage and hospitalisation, defined as an inpatient admission for 24 hours or more in the past six months, was examined through logistic regression. Multivariable logistic regression included variables associated with insurance coverage and those considered to be associated with hospitalisation, both theoretically and through examining crude odds ratios. The role of urban/rural location was examined through stratified odds ratios and formally tested for effect modification. Lastly, reasons for hospitalisation, type of hospital and length of stay were compared to identify any differences between insured and uninsured women. Throughout, self-reported reasons for recent morbidity and hospitalisation were categorised into illness or symptom groups to the extent possible without clinical reports. Given the large variety of illnesses and hence small category sizes, we present morbidity-related data with descriptive proportions rather than formal statistical tests.

## Results

A total of 1,934 adult female respondents (980 uninsured/954 insured) from the selected sample of 1,960 households were interviewed in the baseline survey. Twenty-six insured women were unavailable, mostly in Ahmedabad city, with no replacement available in the same cluster. Demographic and socioeconomic characteristics are presented in Tables 2 and 3.

**Table 2:** Household level characteristics of insured and uninsured women in urban and rural Gujarat (n = 1,934)

	**Rural**					**Urban**				
	**Uninsured**		**Insured**			**Uninsured**		**Insured**		
	**n**	**%**	**n**	**%**	**p value**	**n**	**%**	**n**	**%**	**p value**
*Household structure*										
Extended family	248	44.2	237	44.9	0.76	152	35.3	157	39.6	0.32
Nuclear	312	55.8	321	55.1		268	64.7	239	60.4	
*Mean annual income (INR)*										
0-60,000	258	45.7	273	47.9	0.48	178	39.6	144	34.6	0.16
60,001-120,000	227	40.9	218	39.6		185	43.3	199	54	
120,001-180,000	43	7.5	44	8.5		40	11.8	31	7.6	
180,000+	32	5.9	23	4		17	5.2	22	3.8	
*Dwelling type*										
Mud house	110	21.1	107	23.2	0.70	26	4.8	16	3.0	0.46
Semi	336	57.3	330	55.9		258	54.6	218	52.9	
Solid	114	21.6	121	21		136	40.6	162	44.0	
*Latrine*										
Yes	251	48.6	223	44.7	0.45	297	79.7	299	78.5	0.82
No	309	51.4	335	55.3		123	20.3	97	21.5	
*Religion*										
Hindu	519	89.4	523	92.0	0.70	347	83.3	333	84.6	0.87
Muslim	41	10.6	34	7.8		71	16.2	60	15.0	
*Drinking water*										
Individual tap	415	71.9	406	74.4	0.76	331	85.1	310	82.2	0.64
Shared tap	38	5.7	35	7.0		52	7.8	46	8.7	
Other	107	22.4	117	18.6		37	7.1	40	9.1	

**Table 3:** Individual characteristics of respondents (female respondents ≥15 yrs) n = 1,934

	**Rural**					**Urban**				
**Respondent characteristics**	**Uninsured n = 560**		**Insured n = 558**		**p value**	**Uninsured n = 420**		**Insured n = 396**		**p value**
	**n**	**%**	**n**	**%**		**n**	**%**	**n**	**%**	
*Age group*										
Age 15-24	102	17.3	48	8	<0.001	63	14.7	31	8.5	<0.01
Age 25-34	215	38.7	200	35.8		153	33.7	99	24.3	
Age 35-44	166	28.5	208	37.3		117	27.7	152	38.4	
Age 45-54	60	11.9	83	14.6		64	14.8	85	21.7	
Age 55+	17	3.6	19	4.3		23	9.0	29	7.0	
*Education*										
Never studied	308	51.5	353	62.7	0.02	202	43	184	44.3	0.79
Primary (1–5)	99	18.3	92	18.2		82	18.3	76	20.6	
Secondary+	153	30.2	113	19.1		136	38.6	136	35.2	
*Marital status*										
Married	530	94.4	495	88.2	<0.01	366	86	311	79.5	0.03
Unmarried/divorced	5	0.8	1	0.3		16	4.2	19	4.6	
Widowed	25	4.8	62	11.5		38	9.8	66	15.8	
*Primary occupation*										
Agriculture/Livestock	384	64.4	416	75.9	0.15	11	1.7	8	1.7	0.001
Self-employed/service	71	14.7	74	13.6		201	52.1	255	68.5	
Salaried worker	11	2.4	4	0.7		8	1.8	26	5	
Unemployed	94	18.5	64	9.8		200	44.5	107	24.8	
*Reported 30 day morbidity*										
No	508	89.6	477	85.6		347	83.6	310	79.6	
Yes	52	10.4	81	14.4	0.17	73	16.4	86	20.4	0.36
*Own health perception*										
Poor	10	1.6	12	2.3	<0.01	11	3.1	8	2.4	0.04
Average	413	71.3	434	77.2		264	62.8	288	71.2	
Very good	137	27.1	112	20.5		145	34.1	100	26.5	

### Insurance coverage

Unadjusted odds ratios indicated no major differences in demographic or socioeconomic characteristics at the household level between insured and uninsured respondents, examined separately within rural (n = 1,118) and urban (n = 816) strata (Table 2). Living conditions appeared to vary across location: more rural households lived in precarious mud houses rather than brick or cement dwellings, and urban households were more likely to have a toilet. With respect to individual-level characteristics (Table 3), insured women were older, more likely to be employed, and, in rural areas, less educated than their uninsured counterparts. Also, a higher proportion of insured women were widows. Although insured and uninsured women reported similar levels of morbidity in the past 30 days, insured women were more likely to perceive their own health as average, compared to uninsured women who reported higher levels of very good health.

Multivariable regression (Table 4) indicated similar patterns of demographical characteristics to those observed in the preliminary analysis above. There was no evidence of an association between insurance coverage and reported 30-day morbidity, and the adjusted analysis indicated that average (compared to very good) health status was associated with insurance coverage. There was little evidence that urban/rural location modified the effects of age (p = 0.38) or marital status (p = 0.20) on insurance coverage. There was some evidence that that the association between employment and insurance coverage (p = 0.05) varied with location, with occupation group associated with insurance coverage amongst urban, but not rural, women.

**Table 4:** Factors associated with insurance status amongst adult women (n = 1,934)

**Variable**	**n**	**OR adjusted**	**95% CI**		**p value**
			**LB**	**UB**	
*Age*					<.001
Age 15-24	244	(b)			
Age 25-34	667	1.4	1.0	1.9	
Age 35-44	644	2.2	1.5	3.1	
Age 45-54	292	2.0	1.3	3.2	
Age 55+	88	1.2	0.4	3.3	
*Marital Status*					<.001
Married	1,703	(b)			
Unmarried/Divorced	41	1.4	0.5	3.7	
Widowed	191	2.0	1.5	2.6	
*Education*					0.52
Never educated	1048	(b)			
Primary level	349	1.0	0.7	1.2	
Secondary level	538	0.8	0.6	1.2	
*Occupation group*					<.001
Agriculture	805	(b)			
Self employed	615	0.9	0.6	1.4	
Salaried	49	1.2	0.6	2.6	
Unemployed	465	0.5	0.3	0.7	
*Reported 30 day morbidity*					0.23
No	1,647	(b)			
Yes	287	1.2	0.9	1.7	
*Own health perception*					
Poor	41	(b)			
Average	1,399	1.1	0.6	1.9	0.04
Very good	44	0.8	0.5	1.5	

(b): Baseline group.

### Morbidity and treatment-seeking

Insured women reported slightly higher prevalence of morbidity in the past 30 days than uninsured women (adjusted OR = 1.2), although this difference may be due to chance (p = 0.25). Fever and other common illnesses comprised the majority of cases of morbidity experienced in the past thirty days, followed by symptoms related to hypertension and asthma. There was some variation in symptoms reported by insured and uninsured women in rural areas, although overall the pattern was similar across insurance status (Table 5).

**Table 5:** Type of morbidity experienced in past 30 days (n = 287)

**Illness type**	**Uninsured n = 123**		**Insured n = 164**		**Total n = 287**	
	**N**	**%**	**n**	**%**	**n**	**%**
Accident/injury	4	3.8	6	3.4	10	4
Body pain	13	12.7	16	8.4	29	12
Cold/cough	17	17.6	11	10.0	28	17
Diarrheal	8	6.7	11	5.0	19	7
Eye	0	0.0	2	0.7	2	0
Fever	43	31.3	55	37.0	98	32
Gastric	6	4.6	17	8.5	23	5
Gynaecological	5	3.4	7	4.0	12	4
Respiratory	2	1.6	5	2.5	7	2
Skin	1	0.5	4	4.0	5	1
TB	1	0.4	1	0.5	2	0
Urinary	0	0.0	5	2.5	5	0
Weakness	0	0.0	3	2.0	3	0
NCD	23	17.6	21	11.5	44	17

**Figure 1:**
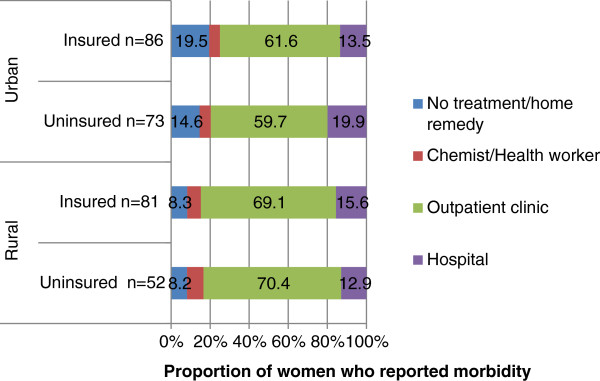
First place of treatment for reported morbidity in the past 30 days.

The majority of women sought treatment in an outpatient clinic setting, with no notable differences in place of first treatment by insurance status (Figure 1). A similar proportion of women sought no treatment or chose to treat themselves with home remedies, with a slightly higher proportion amongst the insured in rural areas. Reported treatment outcomes for recent morbidity were similar for insured and uninsured women (Table 6).

**Table 6:** Treatment result (n = 287)

	**Rural n = 130**				**Urban n = 157**			
	**Uninsured**		**Insured**		**Uninsured**		**Insured**	
**Treatment result**	**n**	**%**	**n**	**%**	**n**	**%**	**n**	**%**
Cured	23	45.3	33	45.7	36	52.9	39	44.4
Not cured*	8	18.4	14	19.5	19	25.1	21	27.4
Treatment continued	21	36.3	31	34.8	16	22.0	26	28.2

*Not cured but treatment discontinued.

### Hospitalisation and insurance coverage

In an unadjusted analysis of women who reported hospitalisation in the past six months (n = 99) (Table 7), insurance coverage was associated with higher odds of hospitalisation in both rural (OR = 2.76, p = 0.001) and urban (OR = 2.45, p = 0.04) women. Amongst the rural insured, average perceived health status (rather than poor or very good) was associated with hospitalisation.

**Table 7:** Hospitalisation amongst rural and urban adult respondents (n = 1,934)

	**Rural**						**Urban**					
	**Uninsured n = 560**			**Insured n = 558**			**Uninsured n = 420**			**Insured n = 396**		
	**n**	**%**	**P value**	**n**	**%**	**p value**	**n**	**%**	**p value**	**n**	**%**	**p value**
Total hospitalised	18	3.2	(b)	46	8.4	0.001	11	3.0	(b)	24	7.0	0.04
*Age group*												
Age 15-24	1	1.4	0.46	4	6.9	0.19	3	5.7	0.61	1	2.7	0.85
Age 25-34	9	4.2		22	11.3		4	3.9		5	3.8	
Age 35-44	6	4.2		15	7.9		3	1.9		11	11.6	
Age 45-54	1	0.7		4	4.5		1	2.2		5	4.3	
Age 55+	1	2.5		1	5.5		0	0		2	6.9	
*Education*		3.8		27	8.1		3	2		10	6.2	
Never studied	13	3.8	0.78	27	8.1	0.90	3	2	0.30	10	6.2	0.09
Primary (1–5)	2	2.1		9	9.9		2	1.6		7	9.8	
Secondary +	3	3.0		10	8.2		6	4.8		7	6.5	
*Marital status*												
Married	17	3.3	0.59	43	8.7	0.60	10	3.1	0.32	21	8.2	0.20
Unmarried/divorced	0	0		0	0		1	7.8		1	5	
Widowed	1	1.9		3	6.1		0	0		2	1.8	
*Primary occupation*												
Self employed	4	6.4	0.69	5	5.8	0.58	8	4.5	0.13	14	6.3	0.30
Agriculture	12	2.9		35	8.8		0	0		0	0	
Salaried worker	0	0		1	20.8		0	0		3	12.3	
Unemployed	2	2.3		5	8.3		3	1.4		7	8.3	
*Mean annual income (INR)*												
0-60,000	11	3.6	0.72	21	8.3	0.54	4	2.2	0.37	10	5.9	0.76
60,001-120,000	6	3.6		17	7.9		3	1.9		11	7.3	
120,001-180,000	0	0		6	13		3	9.8		1	8.7	
180,000+	1	1.5		2	5.8		1	2.6		2	10.7	
*Dwelling type*												
Mud house	4	2	0.54	11	9.1	0.96	0	0	0.39	1	6.7	0.47
Semi	10	2.9		25	8.4		9	3.9		11	5.5	
Solid	4	5.3		10	7.9		2	2.2		12	8.9	
*Reported 30-day morbidity*												
No	18	3.6	0.08	38	8.0	0.42	9	2.9	0.87	19	7.1	0.98
Yes	0	0		8	10.9		2	3.4		5	6.9	
*Own health perception*												
Poor	0	0	0.69	1	0.22	0.03	1	0.0	0.82	0	0	0.62
Average	15	2.6		43	7.8		6	1.7		20	5.3	
Very good	3	0.67		2	0.35		4	1.2		4	1.8	

**Figure 2:**
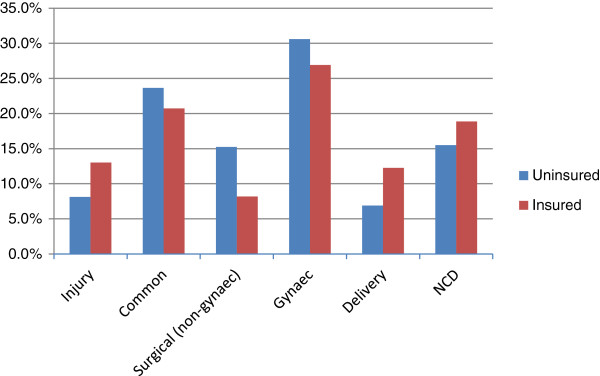
Causes of hospitalisation (% of 70 insured and 29 uninsured hospital cases).

In an analysis adjusted for age, education, marital status, occupation, income group, reported 30-day morbidity and perceived health status, (Table 8), there was strong evidence of an association of insurance coverage with higher odds of hospitalisation (OR = 2.7; 95% ci. 1.6, 4.7). There was no evidence that the association between insurance and odds of hospitalisation varied between urban and rural populations (p = 0.86). No other predictors of hospitalisation emerged.The most common reason for hospitalisation was gynaecological ailments, of which hysterectomy comprised 26 of 31 cases. The mean age of hysterectomy was 36 years. Common ailments such as diarrhoea, fever and vomiting accounted for almost a quarter of cases, followed by non-communicable diseases (Figure 2).

**Table 8:** Association of insurance coverage with hospitalisation (n = 1,934)

**Adult women ≥15 yrs**	**n = 1,934**	**Adjusted OR**	**95% CI**		**p value**
			**LB**	**UB**	
*Insurance status*					0.001
Uninsured	980	(b)			
Insured	954	2.7	1.6	4.7	
*Age*					
Age 15-24	244	(b)			0.62
Age 25-34	667	1.4	0.4	5.3	
Age 35-44	643	1.2	0.4	3.6	
Age 45-54	292	0.6	0.1	2.8	
Age 55+	88	0.4	0.1	2.4	
*Education*					
Not educated	1047	(b)			0.60
Primary	349	0.6	0.2	1.9	
Secondary	538	1.0	0.5	2.1	
*Marital status*					
Married	1,702	(b)			0.31
Unmarried/Divorced	41	2.0	0.4	10.8	
Widowed	191	0.4	0.1	1.7	
*Occupation*					
Self-employed	615	(b)			0.11
Agriculture	805	0.5	0.2	1.3	
Salaried	49	0.3	0.1	1.0	
Unemployed	465	0.4	0.1	1.1	
*Mean annual income(INR)*		(b)			
0-60,000	853				0.72
60,001-120,000	829	0.9	0.4	1.8	
120,001-180,000	158	1.8	0.5	7.2	
180,000+	94	0.8	0.2	3.0	
*Reported 30 day morbidity*					
No	1,642	(b)			0.30
Yes	292	0.6	0.2	1.6	
*Own health*					
Poor	41	(b)			
Average	1,399	2.1	0.3	13.9	0.54
Very good	494	1.4	0.2	9.7	

Table 9 presents risk of hospitalisation by cause. The increased level of hospitalisation in insured women was not concentrated in a particular type of ailment or procedure; insured women appeared to have a higher risk of being hospitalised across a wide range of causes, although numbers were small for individual causes. Insured women were also more likely to be hospitalised for institutional delivery, a service not covered by VimoSEWA.

**Table 9:** Risk of hospitalisation by cause (n = 1,934)

**Cause of hospitalization**	**Uninsured (n = 980)**		**Insured (n = 954)**	
	**n**	**%**	**n**	**%**
Not hospitalized	951	96.9	884	92.2
Injury/Accident	3	0.3	9	1.1
Gastroenteritis, fever	5	0.7	17	1.7
Surgical (non-gynaecological)	3	0.5	6	0.7
Gynaecological including hysterectomy	12	1.0	19	2.2
Childbirth	3	0.2	6	0.6
Non-communicable	3	0.5	13	1.5

Conditional upon having been hospitalised (n = 99), the insured were slightly more likely to stay in the hospital for more than one day, with no difference in length of stay for common illnesses (n = 22) such as fever and diarrhoea (p = 0.49) or if an additional category of two days or more is included (Tables 10). Insurance coverage appeared to affect the choice of where to be hospitalised: the insured used a mix of trust, public and private hospitals, while close to two-thirds of the uninsured used private hospitals, with no use of trust hospitals (Table 11).

**Table 10:** Length of Stay amongst those hospitalised (n = 99)

	**Uninsured n = 29**		**Insured n = 70**	
	**n**	**%**	**n**	**%**
*All illness*				
1 day	8	32.6	17	22.4
>1 day	21	67.4	53	77.6
*Common illness*				
1 day	1	23.8	6	28.7
>1 day	4	76.2	11	71.2

**Table 11:** Place of hospitalisation (n = 99)

**Type of hospital**	**Uninsured n = 29**		**Insured n = 70**	
	**n**	**%**	**n**	**%**
Public	11	36.1	17	19.7
Private	18	63.9	39	58.5
Trust	0	0	14	21.8

## Discussion

This study contributes to the small but growing literature that incorporates an epidemiological approach into the analysis of insurance schemes that cover hospitalisation. While reported morbidity and outpatient treatment-seeking were similar among the VimoSEWA-insured and uninsured, there was strong evidence of higher hospitalisation rates amongst insured women. We interpret our results below and explore what may explain this differential.

### Insurance coverage and treatment-seeking

Demographically, the insured and uninsured were similar in terms of income level, occupation and living standards as indicated by housing/sanitation facilities. The insured comprised slightly older women who were less educated (in rural areas), more likely to be employed, and interestingly, more likely to be widowed than uninsured women. These findings reflect VimoSEWA’s stated goals to reach women workers in the informal economy and those who are vulnerable, such as widows. Lower education levels amongst rural VimoSEWA members compared with their uninsured counterparts contrasts with previous research on the scheme as well as other CBHIs in India, wherein the insured are more likely to be literate [29,31,32]. This survey inquired about formal education levels, rather than literacy, which is typically defined as the ability to sign one’s name. Since SEWA operates literacy programs in rural areas, it is possible that while education is lower, literacy is comparable or higher amongst insured women.

We considered adverse selection – greater likelihood of enrolment by individuals with higher morbidity or proclivity to seek treatment – given previous evidence from voluntary CBHI schemes including VimoSEWA [3,29,33,34]. Although demographic differences in insurance status such as age and marital status may suggest adverse selection, none emerged as independent predictors of hospitalisation in this analysis. The insured and uninsured reported similar recent morbidity rates; VimoSEWA members were not more likely to report a recent illness than uninsured women. Morbidity profiles were also largely similar: common ailments such as fever and body pain comprised the majority of reported illnesses, along with hypertension and diabetes-related episodes for both groups of women. However, uninsured women reported better perceptions of their overall health and this is suggestive of adverse selection. Unfortunately, the cross-sectional nature of our data makes it difficult to arrive at firm conclusions. One complication is that hospitalisation among the insured may itself influence self-reported health and reflect underlying (supply- or demand-side) moral hazard. Self-reported health status may also reflect unobservable attitudes towards treatment-seeking or omitted variables that differ between the insured and uninsured, differences that could be a reason to enrol in – or be a result of – insurance coverage [35,36].

Regarding treatment, insured and uninsured women reported similar first steps after an illness episode in the past month. The majority of women sought care at an outpatient clinic, and the remainder either sought inpatient care or self-treated/did not treat formally in similar proportions. Similar morbidity and outpatient treatment-seeking patterns might lead one to expect that hospitalisation rates would also be comparable amongst the insured and uninsured. Yet we found strong evidence for an association between VimoSEWA coverage and increased odds of hospitalisation amongst adult women in a six-month period. While this finding is consistent with several studies in low-income settings [13,14,37,38], it provides new insight for VimoSEWA in light of earlier research that found no association between VimoSEWA coverage and increased hospitalisation [29].

### Common illnesses

Since VimoSEWA does not cover outpatient care, previous research has suggested that insured women seek hospital-based care in place of outpatient treatment from the outset to avoid out-of-pocket costs [39]. This hypothesis is consistent with a high proportion of claims for common illnesses amenable to outpatient treatment, such as diarrhoea and fever. However, the excess of hospitalisation in insured women was evenly distributed across ailment types. The risk of hospitalisation in the past six months was higher for all causes, not clustered around fever, diarrhoea or other ailments typically treated through outpatient services. Treatment-seeking behaviour for illnesses in the past 30 days did not indicate higher use of hospitals for initial treatment by the insured, including for common illnesses. Reported cure rates were also similar; there was no indication that either group received less effective outpatient care. Further, the length of stay for inpatient hospitalisations – including the proportion of those hospitalised for a 24-hour visit – was similar to that of the uninsured. Insured women were not more likely to be admitted for the minimum one-day period which would qualify for reimbursement. Taken with the opportunity cost associated with hospitalisation for women in the informal sector, this analysis suggests that any substitution of inpatient for outpatient care for common illnesses (a form of moral hazard) by VimoSEWA members may be small.

More insight is provided by recent qualitative research with VimoSEWA-insured urban women who had been hospitalised for fever. Most women indicated that hospitalisation was only sought after outpatient treatment repeatedly failed [40]. Women preferred outpatient care as a first step because it involves lower opportunity costs than hospitalisation – which is consistent with our finding of similar morbidity and outpatient-treatment seeking amongst the insured and uninsured. From insured women’s perspectives, hospitalisation was viewed as a last resort to access more potent treatment. The knowledge that partial costs would be covered by VimoSEWA offered security in the decision-making process. Providers indicated that when they suggest hospitalisation for persistent fever or minor ailments that have become more severe, insured women are more likely to agree. Despite VimoSEWA’s relatively low coverage amounts, this qualitative research suggests that we cannot rule out either provider-induced or demand-side moral hazard.

### Hysterectomy

We examined if the higher hospitalisation rate amongst insured women could be partly explained by higher rates of hysterectomy. Insured women reported slightly higher odds of undergoing a hysterectomy than the uninsured in the past six months, but this difference may be due to chance (p = 0.13). The mean ages at which insured and uninsured women underwent hysterectomy were similar – and relatively young by global standards. The reasons reported for hysterectomy, also similar amongst insured and uninsured women, were gynaecological ailments (fibroids, cysts, menstrual difficulty and to a lesser extent, uterine prolapse) – most of which are amenable to non-invasive, first-line treatment. In a separate analysis of our survey data we found that the proportion of women reporting having ‘ever undergone hysterectomy’ (instead of in a 6-month reference period) was similar between insured and uninsured women, but we did not know their insurance status at the time of the hysterectomy [41].

The data available thus far suggest that having insurance may influence hysterectomy related hospitalisations, possibly as one of a complex set of factors. The coverage provided by VimoSEWA of Rs. 2,000-5,000 covers a significant proportion of the total cost of a hysterectomy (which typically ranges from Rs. 4,000-10,000). Previous qualitative research at SEWA has also identified questionable provider practices, such as conducting hysterectomy on demand or as first-line treatment before less invasive procedures. Provider behaviour is likely to influence the incidence of hysterectomy among women in their mid-thirties – but these practices are likely not limited to insured women or solely in the private sector [41,42]. We are currently exploring the health system and social determinants of hysterectomy through in-depth qualitative research. Initial findings suggest that a high burden of untreated gynaecological morbidity, the lack of primary gynaecological care, treatment practices in both the government and private sectors, and women’s demand for the procedure also contribute to the incidence of hysterectomy in both insured and uninsured women.

### Higher hospitalisation amongst the insured

In the absence of strong evidence that i) having insurance promotes hospitalisation for common illnesses ii) insurance coverage is the primary driver of unnecessary hysterectomy, we explore other possible explanations for higher inpatient admission amongst the insured. One well-established interpretation in the literature is that CBHI is associated with higher utilisation by removing financial barriers to hospitalisation [13-15,38]. This could explain why VimoSEWA coverage was associated with higher odds of hospitalisation in this survey, but not previously in the 2001 analysis by Ranson, when cashless admission facilities were not available. Previous research at VimoSEWA indicated that cashless procedures increases claims submissions overall, but does not improve access to hospitalisation for the poorest [43]^c^.

A second reason for higher hospitalisation may be that the insured have greater knowledge/confidence in negotiating hospitals, resulting in greater utilisation of inpatient care. Fear of navigating complicated hospital admissions procedures has previously been identified as a barrier to both hospitalisation and claims submission, especially amongst the poorest and those living in rural areas [23]. The greater likelihood of seeking hospitalisation could either be a characteristic of insured women, or a result of being insured.

It is possible that women at ease with health services are more likely to enrol in VimoSEWA in the first place. A higher prevalence of institutional deliveries – a service not covered by VimoSEWA – underscores this possibility. Women insured by a CBHI in South India that covers maternity care were twice as likely to deliver in an institution compared to uninsured women [13], while research in three African countries has found that CBHI-insured women do not utilise maternal health services at higher rates if they are not included in the scheme coverage [44]. In this context, it is possible that VimoSEWA membership attracts women more likely to use inpatient services, suggesting adverse selection.

Membership in VimoSEWA itself may result in greater negotiating power. Particularly in the surveyed areas, VimoSEWA members are in continued contact with a concentrated force of grassroots SEWA health and insurance workers who live in the community. CHWs regularly accompany SEWA members (not only the insured) to hospitals and facilitate admission when required – ensuring accessibility to hospitalisation and easier navigation of complicated paperwork, even when procedures are not covered by insurance. In addition to CHWs, VimoSEWA members have the added benefit of dedicated insurance workers. Thus it is plausible that insurance coverage results in women being more able, and perhaps more inclined, to seek inpatient care when required.

Apart from the mechanisms above, insurance coverage may trigger a different decision-making process regarding place and type of treatment, both for women and providers. To illustrate, no uninsured women in the survey population reported use of a non-profit trust hospital, compared to 22 percent of the insured, in the past six months. Since VimoSEWA’s cashless procedures are only available at empanelled hospitals, one-third of which are trust hospitals, the insured are encouraged to seek care at specific institutions. If empanelled providers are incentivised by guaranteed revenue from insured patients, they may provide advice that promotes hospitalisation. Further research is required to explore the treatment decision-making process, and the role, if any, played by moral hazard.

### Study limitations

The research questions addressed arose directly from our experience working with SEWA Health and VimoSEWA; these findings are likely to resonate with both managers and researchers linked to CBHI schemes in low-income settings. Because VimoSEWA’s primary policyholders are women, we did not assess gender differentials or the effect of a women-centred scheme on rationing of health care within the household. As in most household surveys, our analysis is limited by a reliance on self-reported morbidity [35,36]. We confirmed that the pattern of hospitalisation reasons reported by insured women matched that of the VimoSEWA claims database. However, if rates of self-reported morbidity were inaccurate, our analysis may have masked an association of recent morbidity with insurance coverage. Accordingly, our understanding of the pathways associated with greater hospitalisation amongst insured women would change.

If insured women had better recall of hospitalisation in the past six months due to interaction with VimoSEWA, we may have underestimated, or inaccurately categorised reasons for, hospitalisation amongst the uninsured. Lastly, this cross-sectional analysis was limited by an inability to capture unobservable characteristics or omitted variables that may differ systematically between the insured and uninsured; our findings may be biased accordingly.

## Conclusion

From the perspective of a CBHI, increased hospitalisation across a wide range of conditions may reflect the mission to increase access to care. From a public health perspective, however, our findings are of concern. Why is hospitalisation for fever, diarrhoea and gastroenteritis amongst adult women common in the first place? Poor sanitation and limited preventive health practices result in widespread, persistent waterborne ailments. The failure of outpatient care, as indicated by qualitative findings, eventually leads insured women who can seek hospitalisation to do so, in hopes of more effective treatment. In this scenario, insurance appears to compensate for weaknesses in the health system, albeit at a cost to women. Without preventive health measures and quality outpatient care, these illness patterns are likely to persist – and should be of concern both to health policymakers and CBHIs.

Similarly, hysterectomy amongst insured and uninsured women in their mid-thirties is symptomatic of major gaps in the health system, as well as attitudes towards intervention in women’s bodies. Lack of gynaecological care at the primary level, poor knowledge of side effects, provider attitudes that encourage intervention and sociocultural factors all likely play a role in promoting hysterectomy as a common, first-line gynaecological treatment. While insurance, particularly packages with larger coverage than VimoSEWA may facilitate medically unnecessary hysterectomies, the comparable prevalence in uninsured women calls attention to the lack of reproductive health care and the underlying determinants of women’s health in general.

Lastly, if the insured indeed enjoy better access to treatment, it is unclear whether they also enjoy higher quality health care or better health outcomes than those without health insurance. Based on our findings, morbidity patterns and outpatient care were similar up until the point of hospitalisation – but there is no indication of whether higher inpatient admission results in better long term health. Thus far, evaluations of CBHI as well as larger social insurance schemes have focused on the quantitative increase in utilisation and financial security afforded by coverage, with limited assessment of the associated effects on health [2,3,12]. Encouragingly, a recent study in Burkina Faso has investigated the association of CBHI coverage with mortality outcomes [45]. As publicly-funded health insurance schemes such as Rashtriya Swasthya Bima Yojana (RSBY) expand in India and other developing countries, population health needs, access to quality primary care and longitudinal, health outcomes research deserve consideration in program and evaluation design.

## Endnotes

^a^The survey collected information on other types of medical insurance in the household. One household of 1,934 surveyed reported purchase of health insurance outside of VimoSEWA.

^b^The sample size for the household survey was determined based on the trial’s secondary outcome of reduction in hospitalisation for the three focus conditions (diarrhoea, fever and hysterectomy). The primary outcome is reduction in claims as measured by the claims database. For the secondary outcome, based on a between cluster coefficient *k* of 0.28, a sample of 35 uninsured and 35 insured households per each cluster allows for 74% power (p < .05, 2 sided test) to detect a 40% reduction in hospitalisation for diarrhoea, fever and hysterectomy.

^c^Until 2005, VimoSEWA reimbursed members for hospitalisation expenses incurred. In January 2006, VimoSEWA introduced ‘cashless hospitalisation’ where by expenses of hospitalized members were paid directly by VimoSEWA to the hospital.

## Competing interests

The authors declare that they have no competing interests.

## Authors’ contributions

SD and AM conceived the study, and participated in its design and implementation. SD performed the statistical analysis and drafted the manuscript. TS participated in the study design and implementation. SC guided the statistical analyses and drafting of the manuscript. All authors read and approved the final manuscript.

## Pre-publication history

The pre-publication history for this paper can be accessed here:

http://www.biomedcentral.com/1472-6963/14/320/prepub
